# The topical study of inhaled drug (salbutamol) delivery in idiopathic pulmonary fibrosis

**DOI:** 10.1186/s12931-018-0732-0

**Published:** 2018-02-06

**Authors:** Omar S. Usmani, Martyn F. Biddiscombe, Shuying Yang, Sally Meah, Eunice Oballa, Juliet K. Simpson, William A. Fahy, Richard P. Marshall, Pauline T. Lukey, Toby M. Maher

**Affiliations:** 1 Airways Disease Section, National Heart and Lung Institute, Imperial College London and Royal Brompton Hospital, London, UK; 2 Nuclear Medicine Department, Royal Brompton Hospital, Sydney Street, London, UK; 3 GlaxoSmithKline R&D, Clinical Pharmacology, Modelling and Simulation, Stockley Park, London, UK; 4 GlaxoSmithKline R&D, Respiratory Discovery Medicine, Stockley Park, London, UK; 5 GlaxoSmithKline R&D, Fibrosis and Lung Injury Discovery Performance Unit, Stevenage, UK; 6 NIHR Respiratory Biomedical Research Unit, Royal Brompton Hospital, London, UK; 7 Fibrosis Research Group, Inflammation, Repair & Development Section, National Heart and Lung Institute, Imperial College, Sir Alexander Fleming Building, London, SW7 2AZ UK

**Keywords:** Idiopathic pulmonary fibrosis (IPF), Inhaled drug delivery, Gamma scintigraphy

## Abstract

**Background:**

Our aim was to investigate total and regional lung delivery of salbutamol in subjects with idiopathic pulmonary fibrosis (IPF).

**Methods:**

The TOPICAL study was a 4-period, partially-randomised, controlled, crossover study to investigate four aerosolised approaches in IPF subjects. Nine subjects were randomised to receive ^99m^Technetium-labelled monodisperse salbutamol (1.5 μm or 6 μm; periods 1 and 2). Subjects also received radio-labelled salbutamol using a polydisperse nebuliser (period 3) and unlabelled salbutamol (400 μg) using a polydisperse pressurized metered dose inhaler with volumatic spacer (pMDI; period 4).

**Results:**

Small monodisperse particles (1.5 μm) achieved significantly better total lung deposition (TLD, mean % ± SD) than larger particles (6 μm), where polydisperse nebulisation was poor; (TLD, 64.93 ± 10.72; 50.46 ± 17.04; 8.19 ± 7.72, respectively). Small monodisperse particles (1.5 μm) achieved significantly better lung penetration (mean % ± SD) than larger particles (6 μm), and polydisperse nebulisation showed lung penetration similar to the small particles; PI (mean ± SD) 0.8 ± 0.16, 0.49 ± 0.21, and 0.73 ± 0.19, respectively. Higher dose-normalised plasma salbutamol levels were observed following monodisperse 1.5 μm and 6 μm particles, compared to polydisperse pMDI inhalation, while lowest plasma levels were observed following polydisperse nebulisation.

**Conclusion:**

Our data is the first systematic investigation of inhaled drug delivery in fibrotic lung disease. We provide evidence that inhaled drugs can be optimised to reach the peripheral areas of the lung where active scarring occurs in IPF.

**Trial registration:**

This trial was registered on clinicaltrials.gov (NCT01457261).

**Electronic supplementary material:**

The online version of this article (10.1186/s12931-018-0732-0) contains supplementary material, which is available to authorized users.

## Background

Idiopathic pulmonary fibrosis (IPF) is a chronic progressive lung disease associated with a high mortality and a median survival of 3–5 years [1–3]. Two recently approved oral medicines, pirfenidone (Esbriet®) and nintedanib (Ofev®), slow deterioration in lung function, but are associated with side-effects which limit their tolerability [4]. The inhaled drug delivery route offers the advantage of local deposition, smaller drug dose and reduced systemic exposure [5]. However, while this route is used for the treatment of obstructive airway diseases, it is not known whether inhaled drug delivery is feasible in IPF. The optimal region of the lung for topical drug delivery in IPF is likely to be the distal, subpleural regions [6–8]. It is also anticipated that airway distortion, characterised by traction bronchiectasis, may influence lung airway flow characteristics in IPF [9–11]. Thus, observations in healthy subjects and those with asthma and COPD (where the target of therapy is the proximal airways) cannot easily be extrapolated to IPF. Understanding the relationship between particle deposition and lung penetration is a necessary pre-requisite to the development of future inhaled treatments for IPF.

There are only a few clinical trials of inhaled therapy for IPF that are registered in clinicaltrials.gov. These include re-positioning of approved inhaled therapies for pulmonary hypertension and cough (associated with IPF in some subjects) and inhaled fentanyl (an opioid) for pulmonary rehabilitation. There are two trials of inhaled antifibrotics in IPF; an αvβ6 integrin inhibitor (NCT03069989) and a galectin-3 inhibitor [12]. There are other molecules in development for the treatment of IPF by the oral route, as well as the approved drugs pirfenidone and nintedanib. It is difficult to speculate about their suitability for inhaled delivery as this is influenced by various factors (physico-chemical properties as well as dose to be administered). However, Genoa Pharmaceuticals has completed a $62 million Series A financing round to pursue a Phase 2 clinical trial of Aerodone (inhaled pirfenidone) to treat idiopathic pulmonary fibrosis (IPF).

Particle size and inhalation flow are critical factors in determining the total and regional lung deposition of inhaled aerosols [13]. Using monodisperse aerosols and gamma-scintigraphy Usmani and colleagues showed that airway deposition of technetium-labelled monodisperse particles of aerosolised salbutamol was related to clinical effect in asthmatics [13]. Pharmacokinetics can complement these investigations and assess the relative contribution of pulmonary and gastrointestinal absorbtion in terms of rate of absorption and bioavailability of inhaled drug. The amount of salbutamol eliminated in the urine during the first 30 min after inhalation, represents the fraction of drug delivered solely to the lungs; thus providing a measure of bioavailability through the lung [14, 15]. This method provides a means of verifying data generated by radio-labeled imaging of deposition pattern.

We therefore assessed the effect of particle size and aerosol dispersity of inhaled salbutamol on lung deposition in subjects with IPF and related the resultant lung deposition patterns to the pharmacokinetic profile. In so doing, we critically investigated the viability of developing inhaled therapies for the treatment of IPF.

## Methods

### Study subjects

Subjects over the age of 40 years with a multi-disciplinary diagnosis of IPF as defined by current international guidelines [16] and confirmed on high resolution computed tomography (HRCT) within 60 days of study entry, were eligible for participation. Exclusion criteria included: co-existing respiratory disease; adverse reaction to ß2-adrenergic receptor agonists; acute respiratory exacerbation within four weeks of study enrolment.

### Study design

**Fig. 1:**
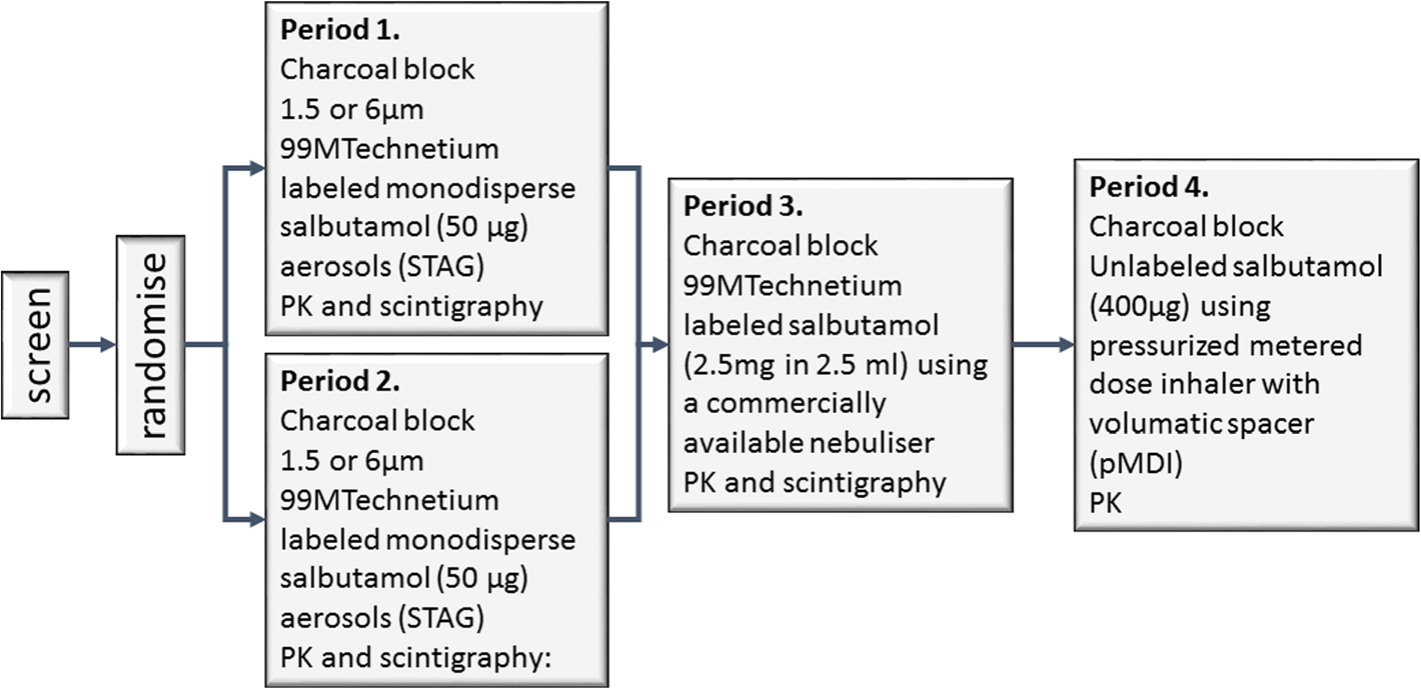
TOPICAL study design. Subjects were randomised to receive either 1.5 or 6 μm ^99m^Technetium labelled monodisperse salbutamol (50 μg) aerosolised using a spinning disk aerosol generator (STAG) in periods 1 and 2. In period 3, all subjects received ^99m^Technetium labelled salbutamol using a commercially available nebuliser. In period 4, all subjects received unlabelled salbutamol via a pressurized metered dose inhaler (pMDI). In all periods, charcoal block was administered prior to dosing with salbutamol

The TOPICAL study (CRT114975; NCT01457261: A s**T**udy **O**f the **P**harmacokinet**IC**s **A**nd deposition of inha**L**ed salbutamol in subjects with idiopathic pulmonary fibrosis) was a Phase Ib, single centre, partially randomised, controlled, 4-period crossover study using technetium labelled salbutamol particles as a tracer molecule to visualise lung deposition in subjects with IPF (Fig. 1).

Subjects were screened and randomised in periods 1 and 2 to receive 50 μg monodisperse technetium-labelled salbutamol at 1.5 μm or 6 μm particle size, such that all subjects received both particle sizes. In period 3 subjects received polydisperse 2.5 mg technetium-labelled salbutamol via nebulisation (PARI BOY SX nebuliser) and in period 4 all subjects received unlabelled polydisperse salbutamol by pMDI with spacer (Volumatic®, Allen & Hanburys, Middlesex, UK). Each treatment period was separated by a minimum of two days and a maximum of two weeks to allow clearance of salbutamol.

Salbutamol was chosen as an ‘inert’ test drug for this study of airway deposition in IPF because of its known safety profile and its known deposition pattern in asthma and COPD. There was no expectation of clinical benefit in IPF [17].

### Particle generation and radiolabelling

The validation, generation and radiolabelling of monodisperse salbutamol (geometric standard deviation (GSD) < 1.22) technetium-labelled salbutamol (GlaxoSmithKline) particles has been described in detail [18, 19]. The spinning-top aerosol generator (STAG Mark II; Research Engineers Ltd.) was used to generate the monodisperse particles. Each dose of radio-labelled salbutamol was delivered as three sequential one-litre bolus breaths followed by a 10 s breath-hold pause.

The polydisperse (GSD > 1.22) nebulised and pMDI plus spacer devices delivered salbutamol (periods 3 and 4, respectively) as standard preparations. Technetium pertechnetate (^99m^TcO_4_^−^) was added to the nebule preparation in the nebuliser chamber. Subjects inhaled the nebulised aerosol as slow and deep tidal breaths which continued until all the liquid was gone. The dose was delivered as four administrations inhaled as slow and deep breaths via the spacer, followed by a breath-hold pause.

### Study procedures

Forced vital capacity (FVC) and diffusion coefficient for carbon monoxide (DLco) were assessed at baseline. Impulse Oscillometry (IOS) was performed at baseline and at 1 and 4 h post-dose. Pulmonary function test (PFT) parameters of slow VC, FEV1, FVC, FEF25–75 and PEF were also measured at baseline and at 1 and 4 h post-dose. Immediately after dosing in periods 1–3 scintigraphic images of the posterior thorax, anterior thorax, and lateral oropharynx were recorded. Blood and urine samples for PK analysis (York Bioanalytical Solutions) were taken before and after dosing in all periods.

Total lung deposition (TLD) and penetration index (PI) were measured by planar gamma scintigraphy imaging and the data processed, as previously described [13, 20].

### Sample size and statistical methods

Based on previous work, a sample size of 8 will have 80% power to detect a difference in means of 0.19 in PI, assuming a standard deviation of differences of 0.15, using a paired t-test with a 0.05 two-sided significance level [12].

Regional lung deposition, TLD and PI were calculated and compared using a linear mixed model method [13]. Interrelationships between disease variables and particle deposition were examined using the Spearman rank correlation coefficient (rho). The Wilcoxon rank sum test was used for unpaired group comparisons of semi-quantitative scales and the Wilcoxon signed rank test for paired comparisons of data. Plasma PK of salbutamol was analysed using non-compartmental analysis (NCA) using WinNonlin Professional Edition version 6 (Pharsight Corporation, Mountain View, CA)]. Urine PK data of salbutamol was summarised as amount of salbutamol excreted in the urine (Ae) by collection window and total over 8 h.

Plasma concentration profiles were normalised to a nominal dose of 1 μg (plasma concentrations divided by the amount of drug administered in each period) to evaluate the relative efficiency of the devices.

To compare PK profiles of salbutamol following pMDI administration in IPF with that observed in healthy and asthmatics, PK profiles were predicted using reported data for healthy subjects [21] and asthmatics [22]. The mean concentration values were extracted from these figures using TechDig (Ronald Jones, TechDig 2.0, Mundelein, IL), and scaled to the pMDI dose (400 μg) administered in this study.

## Results

### Study subjects

Nine IPF subjects were recruited into the study. Mean (± S.D.) age was 66 ± 9 years, seven were males (78%), FVC was 77.32 ± 23.41% predicted, whilst DLco was 51.72 ± 16.71% predicted (Table 1). Although the mean pulmonary function values were relatively well-preserved, the individuals included in the study had a wide range of pulmonary function values with. % predicted DLco ranging from 18% to 78.5% and % predicted FVC from 42% to 117%. Thus, a wide range of pulmonary function was covered. All subjects completed the study except subject number seven, who completed only period 4 (pMDI plus spacer).

**Table 1:** Baseline demographics

Screening Number	Age (Years)	Gender	FVC Absolute (L)	FVC % Predicted	DLCO Absolute (L)	DLCO % Predicted
1	54	Male	2.08	55.3	3.99	46
2	62	Male	3.51	79.6	7.74	78.5
3	70	Female	1.99	85	4.2	61
4	64	Male	2.17	48	3.44	34
5	62	Male	4.36	101	6.38	66
6	78	Female	2.61	117	3.49	52.8
7	70	Male	2.94	79	4.51	53.4
8	81	Male	1.44	42	1.4	18
9	53	Male	3.56	89	5.13	55.8
mean	66		2.74	77.32	4.48	51.72
SD	9		0.88	23.41	1.72	16.71

Subject number 7 completed period 4 only

### Lung deposition

**Fig. 2:**
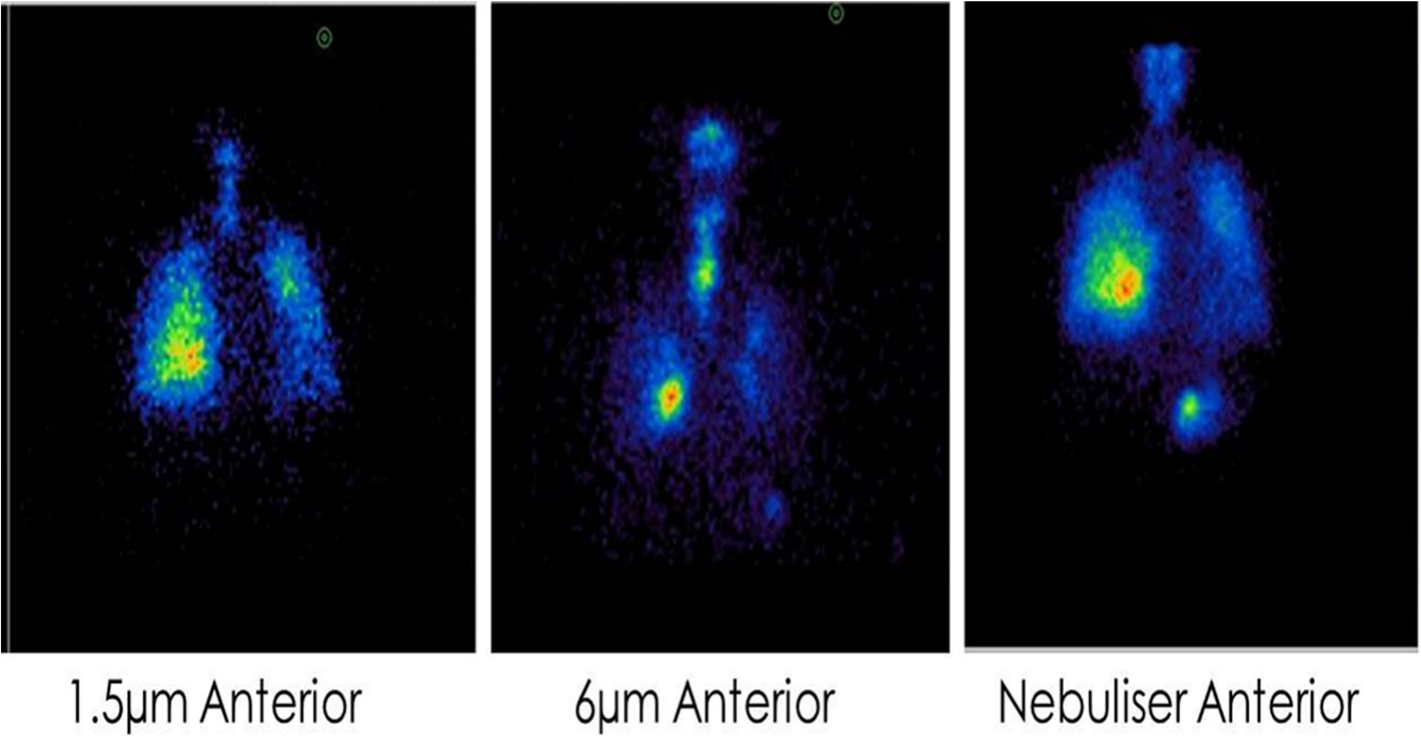
Lung Deposition Images from a representative IPF subject. Anterior thorax γ-camera images of aerosol deposition using technetium-^99m^–labelled salbutamol particles of 1.5 μm, 6 μm mass median aerodynamic diameter (MMAD) using STAG or using a standard nebuliser. Red areas indicate regions of highest radioactivity and black of least radioactivity

**Fig. 3:**
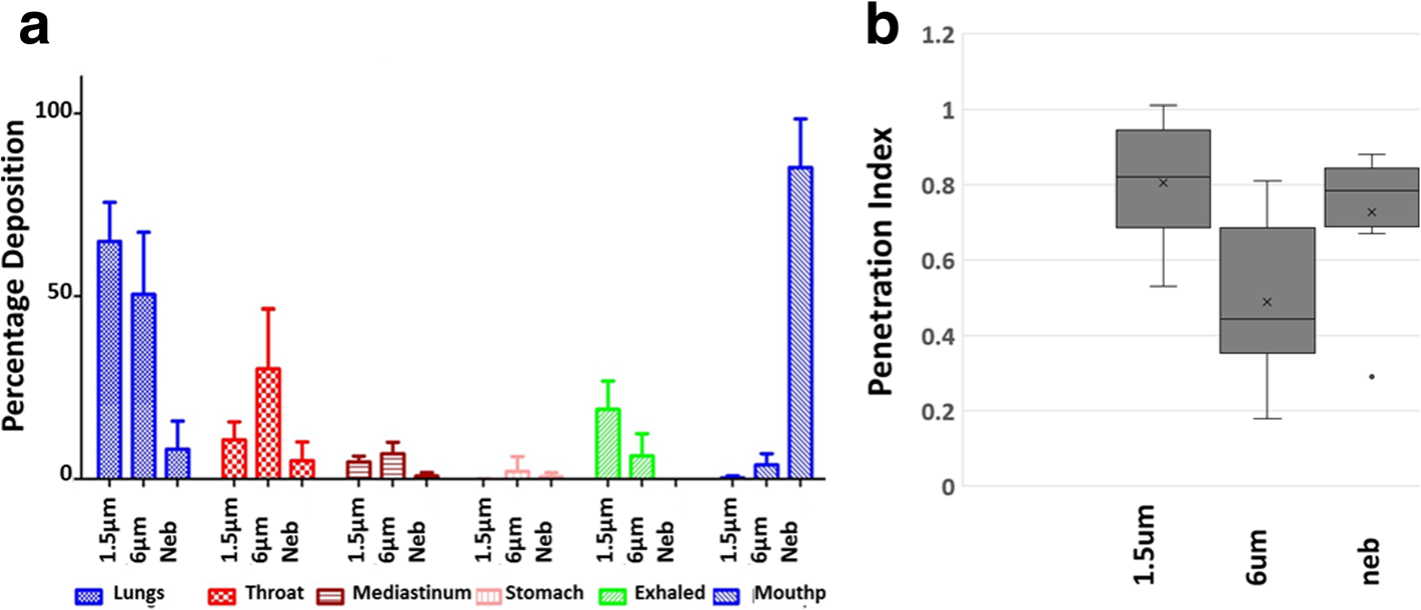
Lung Deposition and penetration Index. (**a**) percentage of total dose deposited in the lungs (*blue*), throat (*red hashed*), mediastinum (*red striped*), stomach (*pink*), exhaled (*green*) and left in the mouthpiece (*blue hashed*) for each delivery method (STAG 1.5 μm and 6 μm and nebuliser). (**b**) Box and whisker plot of lung penetration index. Lung penetration index is illustrated for subjects inhaling the 1.5 μm particle size, the 6 μm particle size and the nebulised salbutamol. Mean (x), median (-), interquartile range (boxes) and outliers (*grey spot*: subject TOP0008) are shown

Lung deposition images were obtained from eight subjects. Representative images for one subject are shown (Fig. 2). 1.5 μm monodisperse particles of salbutamol provided a diffuse pattern of deposition encompassing the peripheral areas of the lung, similar to that generated by the nebuliser (Fig. 2). The 6 μm aerosolised particles concentrated centrally in the throat and airways with less penetration to the periphery (Fig. 2). Mass balance showed the physical distribution of percentage of total dose administered (Fig. 3a).

Monodisperse 1.5 μm particles achieved significantly more efficient TLD, compared to monodisperse 6 μm particles and to the polydisperse nebuliser; (mean ± SD) 64.93% ± 10.72; 50.46 ± 17.04, and 8.19% ± 7.72, respectively *P* < 0.05. However, using polydisperse nebulisation, most of the dose remained in the nebuliser (85.23%; Fig. 3a). The 6 μm particles were deposited in the throat (30.2%) to a greater degree than the 1.5 μm (10.8%) or nebulised salbutamol (5.1%), whereas the 1.5 μm particles (9.1%; Fig. 3a) were more easily exhaled than the 6 μm (6.3%; Fig. 3a). The nebuliser delivered a smaller percentage of drug to the lungs (8.2%) than either of the 1.5 μm (64.9%) and 6 μm (50.5%) monodisperse aerosols (Fig. 3a), although this was compensated for by the much larger initial dose (2.5 mg) delivered by the nebuliser compared to the monodisperse aerosols (50 μg).

The lung PI (mean ± SD) demonstrated that monodisperse 1.5 μm (0.8 ± 0.16) and polydisperse nebulised (0.73 ± 0.19) salbutamol particles penetrated further towards the peripheral areas of the lung than the 6 μm particles (0.49 ± 0.21); (Fig. 3b) (*p* < 0.05). Both right and left lungs achieved largely similar PIs that were not significantly different (*p* > 0.05) (Additional file 1: Figure S6).

Deposition in Inner and Outer ROIs are reported as a percentage of the total dose delivered (mass balance dose) rather than the dose reaching the lung (Additional file 1: Table S5).

**Fig. 4:**
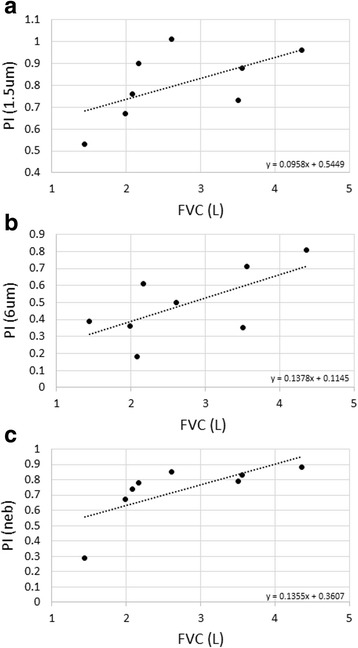
Relationship between pulmonary function and penetration index. Scatter plots of the relationship between FVC (L) and penetration index (PI) for: (**a**) 1.5 μm particle size (STAG), rho = 0.66667 *p* = 0.07099; (**b**) 6 μm particle size (STAG) rho = 0.59524 *p* = 0.11953; (**c**) nebulised rho = 0.92857 *p* = 0.00086. Linear regression analysis (line) is illustrated with its equation. FVC L: forced vital capacity in litres; rho: Spearman Rank Correlation Coefficient

A positive relationship between pulmonary function and PI was identified, such that worsening disease (lower FVC) led to decreased lung penetration in IPF subjects (Fig. 4 a, b and c). This relationship was most marked for FVC (L) and nebulised delivery (rho = 0.92857, *p* = 0.00086, Fig. 4c). The relationship was less clear for other pulmonary function parameters e.g. FVC (%) (rho = 0.80952, *p* = 0.0149) and was absent for absolute DLco (L) (rho = 0.52381, *p* = 0.18272); (see Additional file 1: Figures S8-S10).

### IOS and PFT

There was no difference in the IOS parameters (R5, R20, R5-R20 and X5; Additional file 1: Figure S14) or the PFT parameters (Slow VC, FEV_1_, FVC, FEF_25–75_, PEF; Additional file 1: Figure S15) post-salbutamol dose.

### Pharmacokinetics

Eight subjects provided plasma PK data for monodisperse 1.5 μm (50 μg), 6 μm (50 μg) and polydisperse nebulised (2.5 mg), and all nine subjects provided plasma PK data following 400 μg polydisperse pMDI plus spacer. The plasma PK data is summarised (Table 2) and illustrated (Additional file 1: Figures S7 and S12). Seven subjects provided data for the urine PK analysis (no urine was collected from Subject 1), whilst eight subjects (Subject 7 had urine PK for periods 4) had data for pMDI (Table 3 and Additional file 1: Figure S11). No data were excluded from the PK analyses.

**Table 2:** Summary of PK parameters (AUC(0-t) and Cmax) in plasma by treatment

Variable	Salbutamol Treatment	N	Geo. Mean	CV% Geo Mean	CI 95% Lower GEO Mean	CI 95% Upper GEO Mean
AUC(0-t) pg.h/mL	1.5 μm (50 μg)	8	773.36	38.62	566.23	1056.26
	6 μm (50 μg)	8	1043.94	35.65	781.77	1394.03
	Neb (2.5 mg)	8	4657.29	53.95	3052.22	7106.41
	pMDI (400 μg)	9	1974.04	49.6	1376.59	2830.78
Cmax pg/mL	1.5 μm (50 μg)	8	335.94	41.54	240.65	468.96
	6 μm (50 μg)	8	326.47	39.88	236.79	450.1
	Neb (2.5 mg)	8	1605.95	74.91	919.19	2805.81
	pMDI (400 μg)	9	794.53	34.42	614.34	1027.57

**Table 3:** Summary of urine PK parameters (Amount excreted Ae) by treatment

Interval	Salbutamol Treatment	N	Mean (μg)	Median (μg)	Min (μg)	Max (μg)	Median Percent of Dose (%)
0-30 min	1.5 μm (50 μg)	7	1.5	1.2	0.5	4	3
	6 μm (50 μg)	7	1.2	1	0.7	2.8	2.4
	Neb (2.5 mg)	7	17.4	14.4	0.7	46.3	0.7
	pMDI (400 μg)	8	3.8	4.2	0.9	6.3	1
0-8 h	1.5 μm (50 μg)	6	8.1	6.9	2.7	19.4	16.2
	6 μm (50 μg)	7	12.8	7.6	4.6	29.2	25.6
	Neb (2.5 mg)	7	51.4	64.1	6.7	90.1	2.1
	pMDI (400 μg)	8	16.5	17.6	4.5	28.7	4.1

**Fig. 5:**
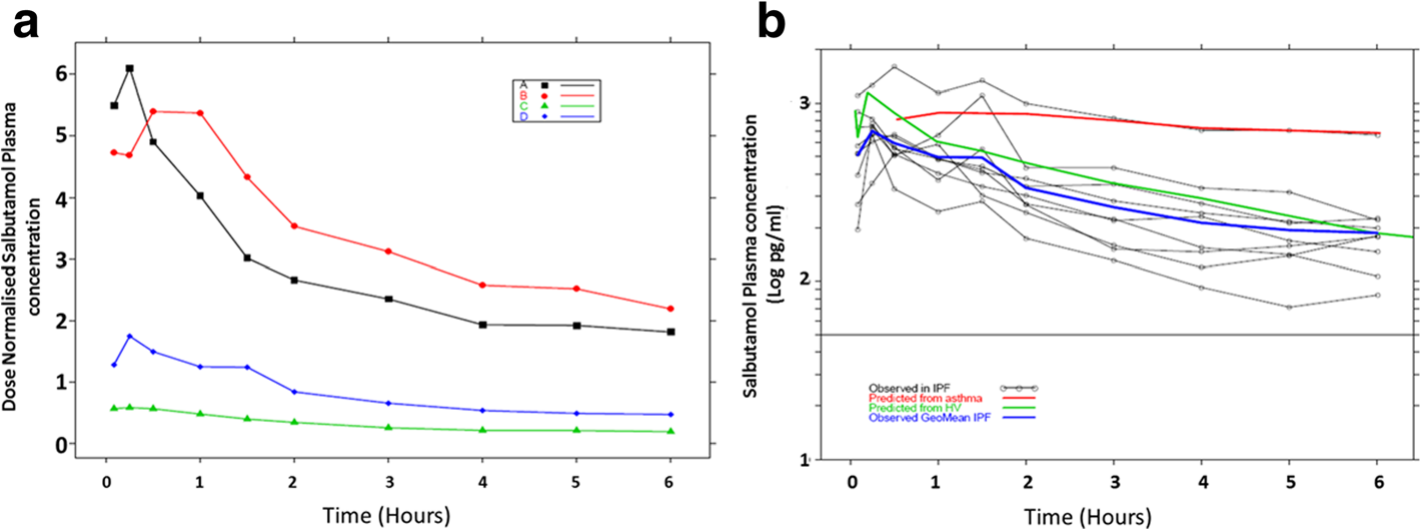
Plasma salbutamol concentrations. (**a**) Dose normalised plasma salbutamol concentration per treatment group. Treatment group 1.5 μm salbutamol 50 μg delivered by the STAG device (■), 6 μm salbutamol 50 μg delivered by the STAG device (●), nebulised 2.5 mg salbutamol (▲), 400 μg salbutamol delivered by the pMDI plus spacer (◊). (**b**) Plasma salbutamol concentration delivered by pMDI plus spacer. Individual subject PK profiles (○) and observed geometric mean PK profile (blue line) for the IPF subjects. Predicted PK profile from asthmatics (red line) [21], predicted PK for healthy subjects (green line) [20]

Dose normalised plasma PK profiles indicate that monodisperse 1.5 μm and 6 μm salbutamol delivered by the STAG device were similar and more efficient at entering the systemic circulation than the other methods (polydisperse nebulised and pMDI); with nebulised delivery achieving lower levels of drug in the systemic circulation (assuming the same dose, nebulised AUC(0-t) was 0.12%, and pMDI was 32% of the AUC delivered by 1.5 μm STAG; Fig. 5a).

Individual PK profiles and the geometric mean PK profile for the IPF subjects using the pMDI plus spacer device, as well as the mean PK profile predicted from healthy subjects [21] and asthmatics [22] data are shown (Fig. 5b). Cmax was similar between the healthy subjects and asthmatics but was somewhat lower in IPF (Fig. 5b). The elimination phase in IPF and healthy subjects was similar but faster than in asthmatics (Fig. 5b). Plasma PK profiles for each treatment group (Additional file 1: Figures S7 and S12) are illustrated.

In terms of percentage of total dose, the amount of salbutamol excreted in urine after 30 min was similar between 1.5 μm and 6 μm STAG, and higher than nebulised delivery, indicating relatively higher lung absorption from the monodisperse devices. However, there was no apparent relationship between the amount of salbutamol deposited in the lungs (Additional file 1: Table S4) and the amount excreted in urine after 30 min (Additional file 1: Figure S13), probably due to the narrow range in lung dose. Individual urine PK profiles for the 1.5 μm and 6 μm treatment groups are illustrated (Additional file 1: Figure S11).

## Discussion

To the best of our knowledge, this is the first study in subjects with IPF to quantify inhaled drug delivery and to determine the optimal aerosol particle size for inhalation. The development of new treatments for IPF has focussed largely on identifying targets that localise to the peripheral and basal areas of fibrotic honeycomb lung [23, 24]. Both air- and blood-flow are disrupted in these areas [9] posing significant challenges to systemic and inhaled drug delivery. The effectiveness of systemic oral delivery has been confirmed in subjects with IPF with both pirfenidone and nintedanib [25–27]. However, the toxicity of systemic therapy remains a challenge in clinical practice with sizable proportions of patients being unable to tolerate long term therapy with available anti-fibrotic drugs. Specifically, in a real-world setting, retention on pirfenidone requires active management and dose adjustments; in the PASSPORT registry of > 1000 subjects with IPF 31% discontinued pirfenidone over 2 years and this is reduced to 20% by dose reduction [28]. Aerosolised drug delivery may provide an advantageous option in IPF. However, the effectiveness of this route has yet to be proven in fibrotic lung disease.

We have shown that 1.5 μm diameter aerosol particle sizes are able to effectively penetrate to the peripheral areas of the lungs of subjects with IPF. Whereas, 6 μm particles deposit centrally in the throat and proximal airways with less penetration to the lung periphery. Whilst similar findings have been reported in healthy and asthmatic subjects [13, 29], data in subjects with IPF, in whom airway flow characteristics are altered due to significant architectural distortion and traction bronchiectasis, has hitherto been lacking. Our data are novel and indicate that despite the effects of fibrotic destruction of the lung, small particles can penetrate to the distal regions in IPF.

The IPF lung demonstrates heterogeneous distribution of fibrosis with apparently normal areas of alveolar tissue abutting active regions of fibroproliferation and end-stage honeycomb fibrosis. It is plausible that the optimal site of action of any drug is at any of three possible sites; 1) in areas of established fibrosis where pro-fibrotic pathways are active, 2) at the leading edge of fibrosis (the fibroblastic foci) where nascent fibrotic tissue appears to be laid down or 3) within structurally normal lung where treatment may prevent the development of fibrosis. The optimal site for drug delivery may vary, depending on the mode of action of a given drug. Nonetheless, this study describes the effect of particle size and quantifies the relative amount of drug deposited peripherally and distally. Drug developers may use this information to optimise the dose administered and the particle size to ensure that the correct amount reaches the target area of the lung which they consider most important. In addition, knowledge of the likely amount deposited centrally will facilitate the establishment of suitable safety limits based on the pre-clinical toxicology. The necessary next step will be demonstration of pharmacodynamic effects of novel compounds in the lungs following inhaled administration. The data from the TOPICAL study can be used to guide the future development of drugs to effectively deliver inhaled aerosol to the target pathological sites in the lungs of subjects with IPF.

Relationships between pulmonary function parameters and lung PI have been demonstrated in some pulmonary diseases [30, 31], although not asthma [13]. In IPF, we show that there is a relationship between pulmonary function and lung deposition for all particle sizes tested; the lower the FVC, the lower the PI. This suggests that although inhaled drugs should be suitable for all IPF subjects, dose and device will require optimisation to ensure adequate dose is delivered to the lung periphery across all severities of disease.

Experience of inhaled drug delivery in IPF is limited to a few opportunistic studies of N-acetylcysteine [32–34], interferon-gamma (IFN-γ) [35, 36] and heparin [8]. These studies were generally inconclusive as to the utility of inhaled drug delivery in the treatment of IPF due to small numbers of subjects and a lack of clinical or safety benefits. Using lung gamma-scintigraphy, Diaz and colleagues observed that inhaled interferon-gamma, delivered using a ‘smart’ efficient nebuliser system (1.7 μm), penetrated to the lung periphery [35]. Whilst no clinical benefit has been described using inhaled delivery in IPF, these results are encouraging for inhaled drug delivery to subjects with IPF; indicating that IPF subjects tolerate nebulised delivery twice per day for an extended period; and that both small (MW ≤ 1000) and large molecules (MW >> 1000) may be delivered. More recently, novel anti-fibrotic therapies are being developed for IPF that are solely to be delivered by the inhaled route (e.g. [12] and NCT02612051). Understanding the deposition in the lungs and absorption into systemic circulation following inhalation in this specific disease is crucial to success of such novel therapies.

Patient compliance may be an issue with inhaled drugs for IPF, however, studies in COPD have indicated that the durability, ergonomics and ease of use of the delivery device were relevant factors in determining compliance; age and breathlessness did not affect compliance (Chrystyn et al. 2014). Optimising the delivery device for IPF is another area of future research. It may be prudent to develop the new medicine as an integral unit that includes the drug and the device.

The pharmacokinetic (PK) profile of subjects with IPF was generally in agreement with that predicted for healthy subjects [21], indicating that despite their disease, IPF subjects had relatively healthy metabolic function (similar clearance). Cmax was slightly lower in IPF subjects, possibly reflecting a reduced lung surface area for absorption. In contrast, compared to PK data in asthmatics [22], the salbutamol PK profile in subjects with IPF exhibited a ‘flatter’ profile. This difference may reflect potential differences in lung β-agonist receptors rather than differences in elimination of salbutamol, as PK is similar following intravenous dosing of salbutamol in asthmatics and healthy subjects [37]. The rapid appearance of detectable salbutamol in the systemic circulation after an inhaled dose in IPF is encouraging, as it suggests significant lung deposition and absorption. This is supported by the gamma scintigraphy where a PI like that observed in asthmatics was observed. In contrast, the AUC data indicated that the aerosol dispersity was important; monodisperse aerosols (GSD < 1.22) of salbutamol delivered by the STAG device had higher AUC values than the polydisperse (GSD > 1.22) nebuliser and pMDI, This suggests that efforts should be made by device engineers and formulation scientists to achieve a narrower dispersion of the inhaled drug.

Systemic availability of inhaled salbutamol results from both oral (swallowed) and pulmonary (inhaled) absorption [38]. The amount of urinary excretion in the first 30 min following inhaled dosing reflects the relative bioavailability of salbutamol absorbed through the lungs with negligible contribution from the oral route [15, 39–42]. Salbutamol exposure, as reflected by the AUC and total urinary excretion, were lower for the 1.5 μm particles compared to 6 μm particles; suggesting that peripheral deposition may result in lower systemic availability. Our data would suggest an advantage for using a smaller drug particle size in subjects with IPF.

Although, a small number of subjects was studied, clear patterns of lung deposition were observed based on particle size and the method of administration. We chose to study only two particle sizes of salbutamol; however, the 1.5 μm and 6 μm particles bracket the ‘respirable range’ and allowed us to test the optimal particle size by studying particles at either end of this range. We did not accurately modulate the inspiratory flow rate of the IPF subjects, a factor which might be expected to modify deposition, particularly as the monodisperse aerosols were inhaled using three sequential one-litre bolus breaths followed by a 10 s breath-hold pause, whereas the conventional polydisperse nebuliser delivery used continual tidal breathing. Finally, the physico-chemical properties of salbutamol are specific to this molecule and extrapolation to other chemical entities should take this into account.

## Conclusion

This study provides important results to guide drug discovery programmes in the future development of inhaled drugs for the treatment of subjects with IPF. The choice of drug particle size diameter, aerosol particle size dispersion, coupled with innovative device technology will be important factors to consider in targeting drug to the distal sites of lung pathology in these subjects. This study confirms the feasibility of inhaled drug delivery in IPF and supports the development of topical therapies for this progressive and ultimately life-shortening disease.

## Additional file

Additional file 1: Particle generation and radiolabelling (DOCX 573 kb)

## Abbreviations

Ae: Excreted in the urine
AUC: Area under the curve
Cmax: Maximum observed concentration
COPD: Chronic obstructive pulmonary disease
DLco: Diffusion coefficient for carbon monoxide
FVC: Forced vital capacity
GSD: Geometric standard deviation
HRCT: High resolution computed tomography
IOS: Impulse Oscillometry
IPF: Idiopathic pulmonary fibrosis
MW: Molecular weight
PFT: Pulmonary function test
PI: Penetration index
PK: Pharmacokinetics
pMDI: Pressurized metered dose inhaler with volumatic spacer
ROI: Region of interest
STAG: Spinning-top aerosol generator
TLD: Total lung deposition
