# Supplementary material for: The topical study of inhaled drug (salbutamol) delivery in idiopathic pulmonary fibrosis
Source: Respir Res. 2018 Feb 6;19:25. doi: 10.1186/s12931-018-0732-0 (PMC5801831; doi:10.1186/s12931-018-0732-0)
Supplement: Additional file 1: — Particle generation and radiolabelling (DOCX 573 kb) [file 12931_2018_732_MOESM1_ESM.docx]

# Supplementary Methods

## Particle generation and radiolabelling

The validation, generation and radiolabelling of monodisperse (geometric standard deviation (GSD) < 1.22) technetium-labelled salbutamol particles has been described in detail [17, 18]. The spinning-top aerosol generator (STAG Mark II; Research Engineers Ltd.) was used to generate the monodisperse particles. Immediately prior to delivery from the STAG, sabutamol sulfate powder (GlaxoSmithKline), dissolved in ethanol, was combined with a measured quantity of technetium pertechnetate (^99m^TcO_4_^-^), eluted from a molybdenum/technetium generator (Mallinckrodt, Radiopharmacy Services, Middlesex Hospital). Each dose was delivered as three sequential one-litre bolus breaths followed by a 10 second breath-hold pause.

The polydisperse (GSD>1.22) nebulised and pMDI plus spacer devices delivered salbutamol (periods 3 and 4, respectively) as standard preparations (Ventolin, Allen & Hanburys, Middlesex, UK). Technetium pertechnetate (^99m^TcO_4_^-^) was added to the nebule preparation in the nebuliser chamber. The nebuliser particles had a mass median aerodynamic diameter (MMAD) 3.1µm with a GSD of 1.8. Subjects inhaled the nebulised aerosol as slow and deep tidal breaths which continued until all the liquid was gone. The pMDI (400µg, HFA  Evohaler^®^) had an MMAD of 2.6µm. The dose was delivered as four administrations inhaled as slow and deep breaths via the spacer, followed by a breath-hold pause.

## Study procedures

Forced vital capacity (FVC) and diffusion coefficient for carbon monoxide (DLco) were assessed at baseline. Impulse Oscillometry (IOS), a sensitive assessment of large and small airways function, was performed at baseline and at 1 and 4 hours post-dose, where total airway resistance at 5 Hz (R5), central airways resistance at 20Hz (R20), small airways resistance (R5 - R20) and small airway reactance measured at 5Hz (X5) were determined. Pulmonary function test (PFT) parameters of slow VC, FEV1, FVC, FEF25-75 and PEF were also measured at baseline and at 1 and 4 hours post-dose. Charcoal block (240mL of water plus 50g activated charcoal) was offered to subjects prior to dosing of salbutamol in all four periods, to minimise gastrointestinal absorption of any swallowed dose. Immediately after dosing in periods 1 – 3 scintigraphic images of the posterior thorax, anterior thorax, and lateral oropharynx were recorded. Blood and urine samples for PK analysis were taken before and after dosing in all periods.

## Imaging

Total lung deposition (TLD) and penetration index (PI) were measured by planar gamma scintigraphy imaging and the data processed, as previously described [13, 19]. Lung regions of interest (ROI) were generated using a Krypton ventilation scan to define the lung boundaries and Inner and Outer ROIs were generated [20]. The deposition in the lungs, oropharynx, mediastinum, stomach and aerosol exhaled and retained in the device were calculated by performing a “mass balance” where, allowing for tissue attenuation, their sum was assumed to be 100%.

## Pharmacokinetics

The pharmacokinetics of salbutamol was measured in plasma and urine (York Bioanalytical Solutions). Human plasma and urine samples were analysed for salbutamol using a validated analytical method based on solid phase extraction for plasma and dilution with buffer for urine, followed by LC-MS/MS analysis.

Blood samples were drawn at baseline (t0), at 5, 15 and 30 minutes, 1, 1.5, 2, 3, 4, 5 and 6 hours post salbutamol. Urine was collected at 0 to 30 minutes, 30 minutes to 4 hours and 4 to 8 hours and aliquots were frozen at -80^o^C prior to analysis.

## Statistical methods

Regional lung deposition, TLD and PI were measured using planar images and calculated as previously described [13]. The mean values of TLD and those of PI were compared using a linear mixed model method.

Penetration Index (PI) is a continuous variable between 0 and 1.0. A value of 1.0 implies complete penetration of aerosol to the peripheral lung boundary. PI allows an accurate assessment of differences in regional lung deposition between different particle sizes and charge states. Based on our previous work, a sample size of 8 will have 80% power to detect a difference in means of 0.19 assuming a standard deviation of differences of 0.15, using a paired t-test with a 0.05 two-sided significance level [12].

Interrelationships between disease variables and particle deposition were examined by using the Spearman rank correlation coefficient (rho). The Wilcoxon rank sum test was used for unpaired group comparisons of semi-quantitative scales and the Wilcoxon signed rank test for paired comparisons of data. Plasma pharmacokinetic data of salbutamol was analysed using non-compartmental analysis (NCA) using WinNonlin Professional Edition version 6 (Pharsight Corporation, Mountain View, CA)]. The following PK parameters were estimated: maximum observed plasma concentration (Cmax), first time to reach Cmax (tmax) and time of the last observed plasma concentration (tlast) and area under the plasma concentration-time curve from time zero to the last quantifiable time point (AUC(0-t)). Urine PK data of salbutamol was summarised as amount of salbutamol excreted in the urine (Ae) by collection window and total over 8 hours. For both PK analyses, the planned time was used.

Plasma concentration profiles were normalised to a nominal dose of 1µg (plasma concentrations divided by the amount of drug administered in each period) to evaluate the relative efficiency of the devices.

To compare PK profiles of salbutamol following pMDI plus spacer administration in IPF with that observed in healthy and asthmatics, PK profiles were predicted using the reported data in the literature, for healthy subjects [21] and asthmatics [22]. The mean concentration values were extracted from these figures using shareware TechDig (Ronald Jones, TechDig 2.0, Mundelein, IL), and then scaled to the pMDI dose (400µg) administered in period four of this study.

## Charcoal Block Results

Two subjects tolerated 240mL of oral charcoal, three subjects did not take any charcoal, the others drank between 10mL and 120mL of charcoal; no apparent relationship between PK and the amount of charcoal consumed was detected. The PK results reported here included all subjects regardless of charcoal intake.

Variable volumes of activated charcoal were tolerated by our IPF subjects, where the majority was unable to ingest the planned full dose. However, the amount of drug excreted in urine during the first 30 minutes after dosing, accurately reflects lung absorbtion and is independent of swallowed dose.

# Supplementary Data

**Table 4: Total lung deposition as a percentage of administered dose for each subject**

| Subject | 1.5µm  50µg | 6µm  50µg | Nebuliser  2.5mg |
| --- | --- | --- | --- |
| 001 | 70.85 | 65.10 | 9.30 |
| 002 | 69.05 | 51.43 | 11.43 |
| 003 | 78.96 | 49.93 | 1.02 |
| 004 | 60.15 | 56.52 | 23.57 |
| 005 | 48.68 | 17.55 | 1.06 |
| 006 | 64.60 | 68.23 | 11.93 |
| 008 | 52.09 | 33.98 | 1.25 |
| 009 | 75.08 | 60.93 | 5.95 |
| Mean | 64.93 | 50.46 | 8.19 |
| SD | 10.72 | 17.04 | 7.72 |

Subject number 007 declined scintigraphy and only completed period 4 (pMDI)

**Table 5: Lung deposition in the inner (I) and outer (O) regions as a percentage of administered dose for each subject**

| Subject | 1.5µm 50µg | | 6µm 50µg | | Nebuliser 2.5mg | |
| --- | --- | --- | --- | --- | --- | --- |
|  | I | O | I | O | I | O |
| 001 | 31.49 | 39.36 | 50.15 | 14.95 | 4.22 | 5.08 |
| 002 | 29.99 | 39.06 | 31.69 | 19.74 | 4.71 | 6.72 |
| 003 | 32.75 | 46.21 | 28.59 | 21.34 | 0.42 | 0.60 |
| 004 | 17.60 | 42.55 | 21.32 | 35.20 | 7.59 | 15.98 |
| 005 | 16.33 | 32.35 | 6.55 | 11.00 | 0.38 | 0.68 |
| 006 | 21.24 | 43.36 | 33.81 | 34.42 | 11.08 | 0.85 |
| 008 | 22.42 | 29.67 | 17.21 | 16.77 | 0.97 | 0.29 |
| 009 | 27.23 | 47.85 | 25.06 | 35.87 | 5.12 | 0.83 |
| Mean | 24.88 | 40.05 | 26.80 | 23.66 | 4.31 | 3.88 |
| SD | 6.36 | 6.37 | 12.85 | 10.02 | 3.76 | 5.45 |

Subject number 007 declined scintigraphy and only completed period 4 (pMDI)


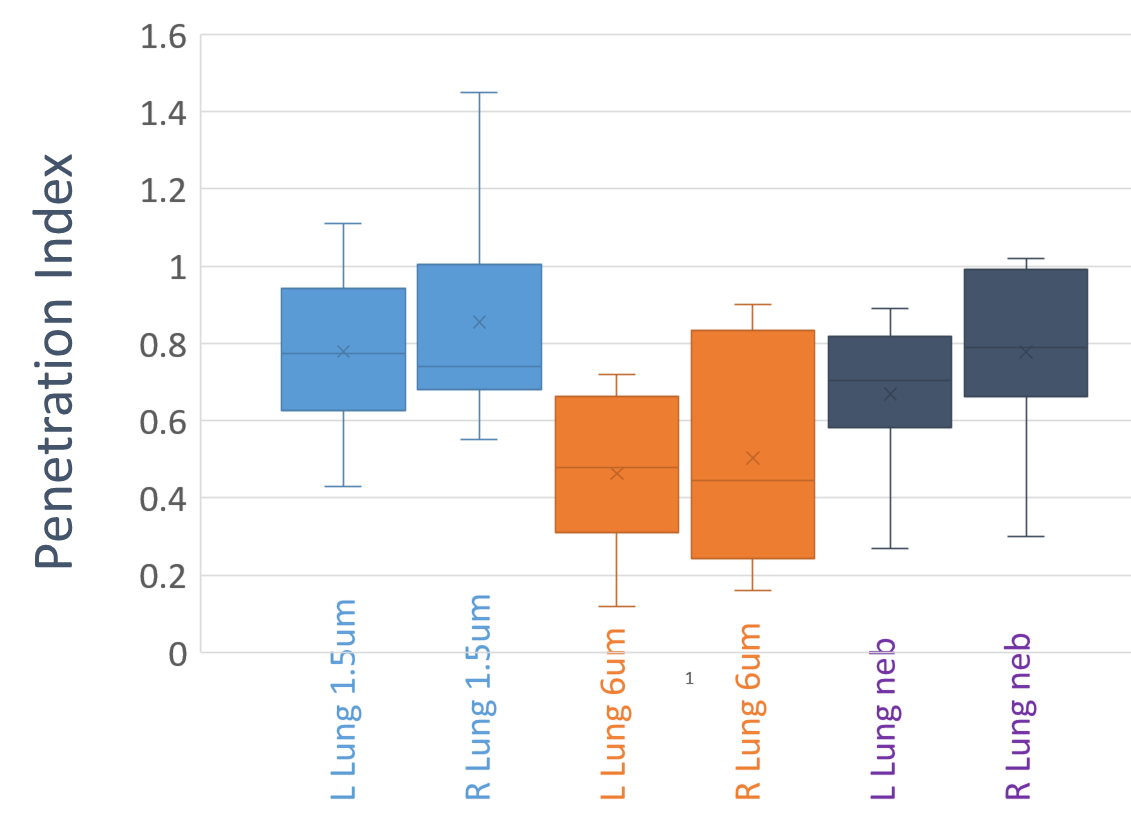


**Figure 6 Box and whisker plot of lung penetration index for subject lungs.** Lung penetration index is illustrated for the left and right lungs separately. Results for the 1.5µm particle size, the 6µm particle size and the nebulised salbutamol are shown. Mean (x), median (-), interquartile range (boxes) are shown.


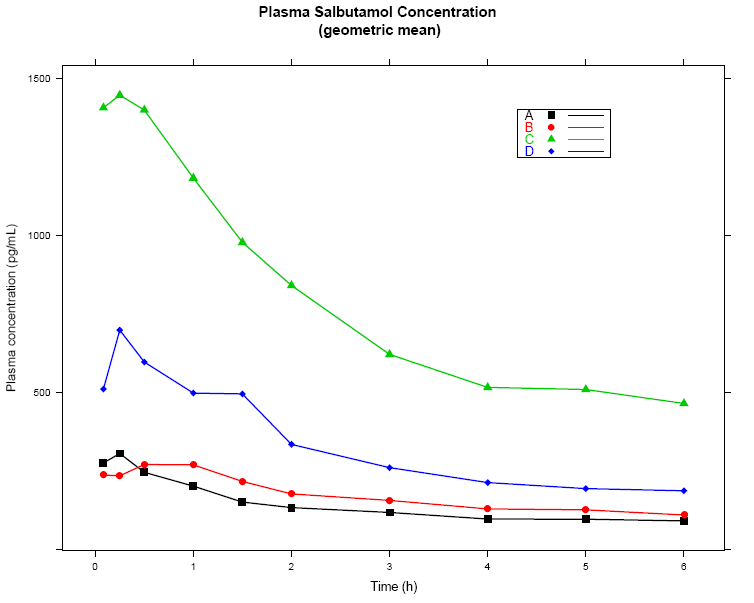


**Figure 7: Plasma salbutamol concentration per treatment group.** Treatment group 1.5µm salbutamol 50µg delivered by the STAG device ( ∎), 6µm salbutamol 50µg delivered by the STAG device ( ●), nebulised 2.5 mg salbutamol ( ▲), 400µg salbutamol delivered by the pMDI ( ◊).


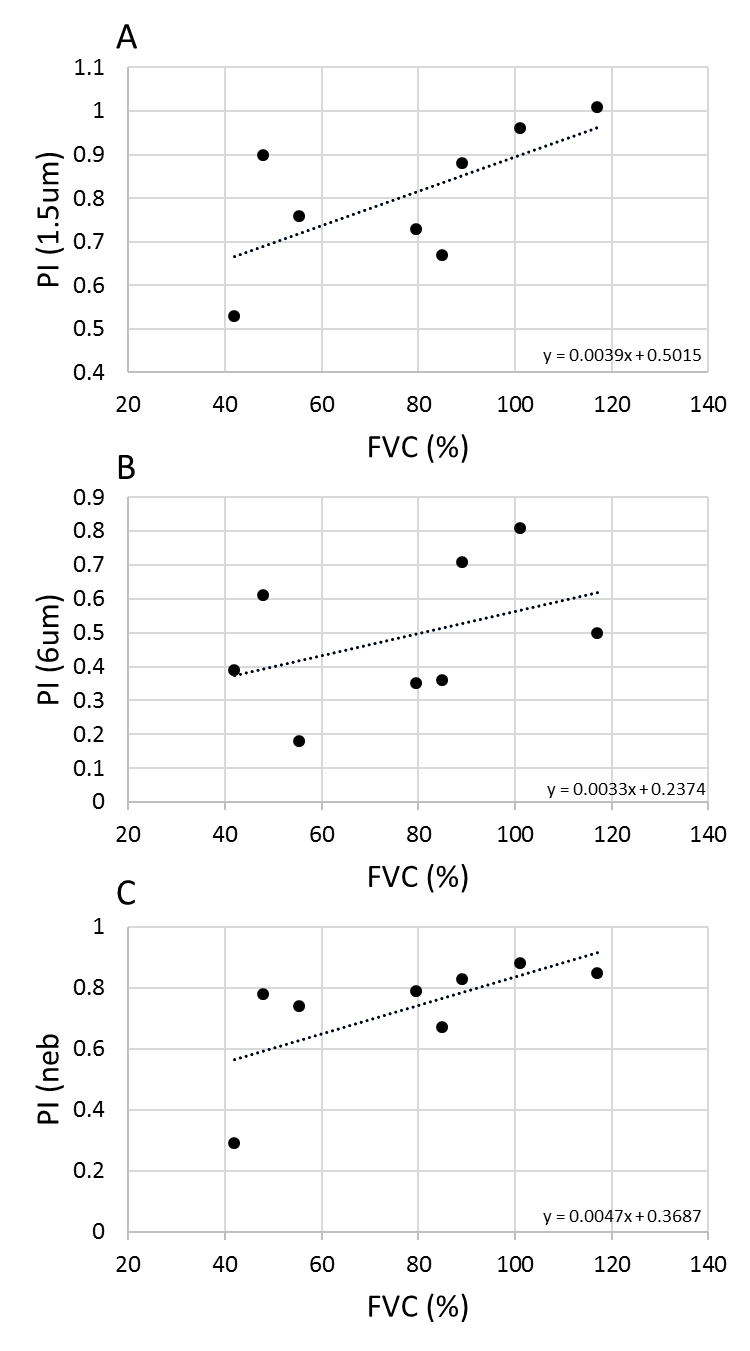


**Figure 8: Relationship between pulmonary function and penetration index**. Scatter plots of the relationship between FVC (%) and penetration index (PI) for: (A) 1.5µm particle size (STAG), rho = 0.66667 p = 0.07099; (B) 6µm particle size (STAG) rho = 0.42857 p = 0.2894; (C) nebulised rho = 0.80952 p = 0.0149. Linear regression analysis (line) is illustrated with its equation. FVC (%): percent predicted forced vital capacity; rho: Spearman Rank Correlation Coefficient


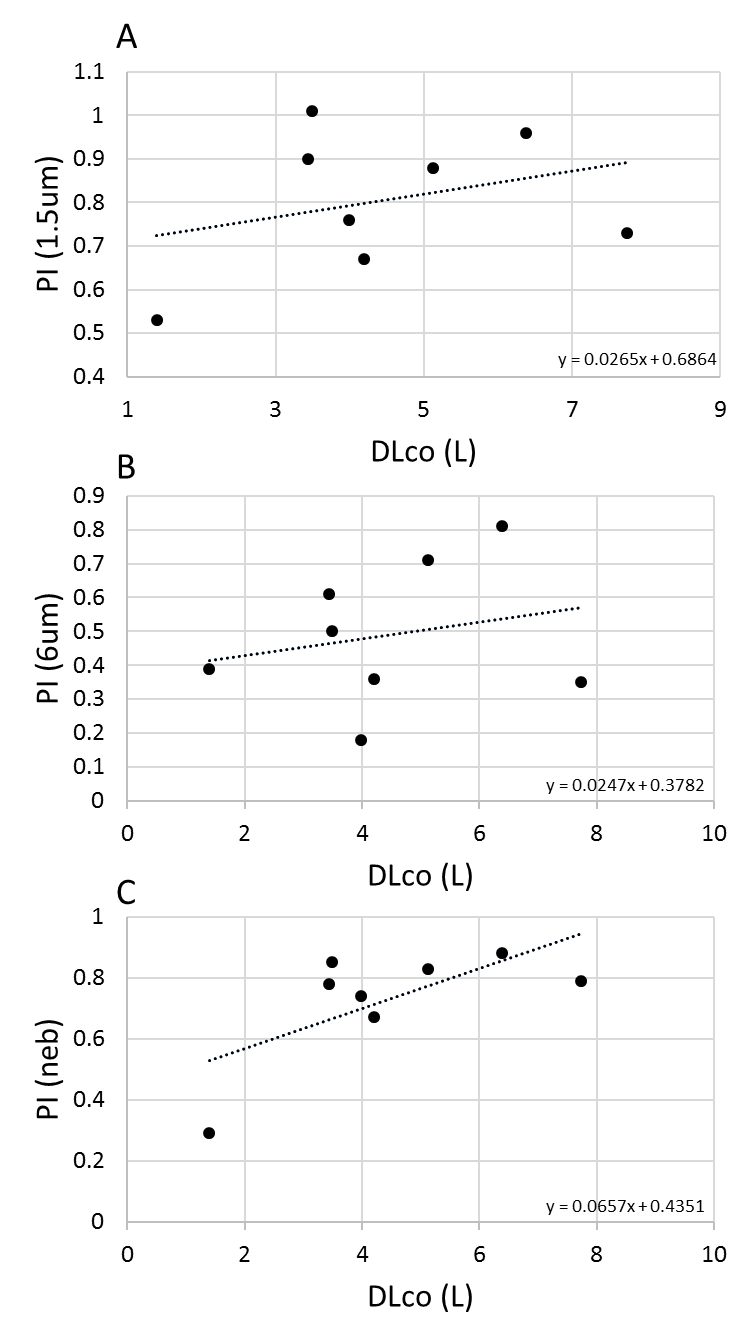


Figure 9 **Relationship between pulmonary function and penetration index**. Scatter plots of the relationship between DLco (L) and penetration index (PI) for: (A) 1.5µm particle size (STAG), rho = 0.09524 p = 0.82251; (B) 6µm particle size (STAG) rho = 0.04762 p = 0.91085; (C) nebulised rho = 0.52381 p = 18272. Linear regression analysis (line) is illustrated with its equation. DLco (L): diffusion capacity for carbon monoxide measured in litres; rho: Spearman Rank Correlation Coefficient


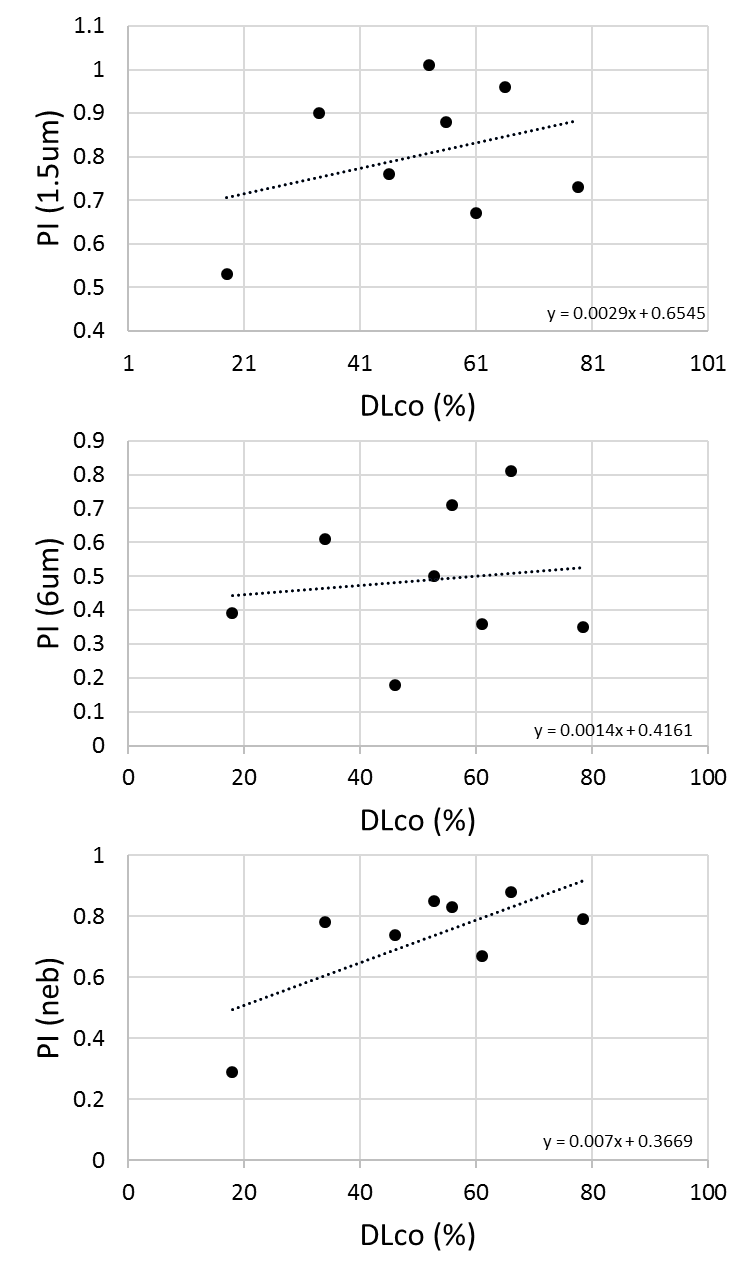


Figure 10 **Relationship between pulmonary function and penetration index**. Scatter plots of the relationship between DLco (%) and penetration index (PI) for: (A) 1.5µm particle size (STAG), rho = 0.11905 p = 0.77889; (B) 6µm particle size (STAG) rho = 0.04762 p = 0.91085; (C) nebulised rho = 0.52381 p = 18272. Linear regression analysis (line) is illustrated with its equation. DLco (%): percent predicted diffusion capacity for carbon monoxide; rho: Spearman Rank Correlation Coefficient


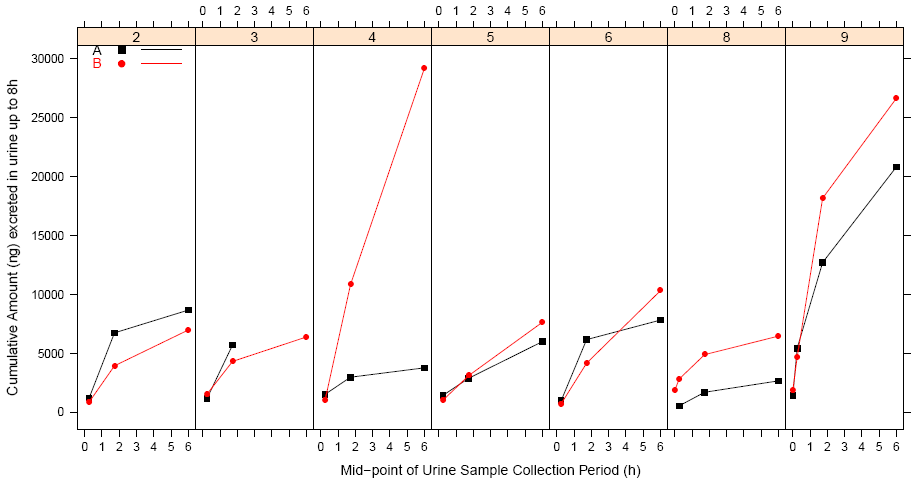


**Figure 11: Salbutamol PK in urine of IPF subjects.** Cumulative levels of salbutamol in the urine of IPF subjects over 8 hours post dose. Salbutamol was delivered at 1.5µm particle size via the STAG device (black line) or 6µm particle size via the STAG device (red line)


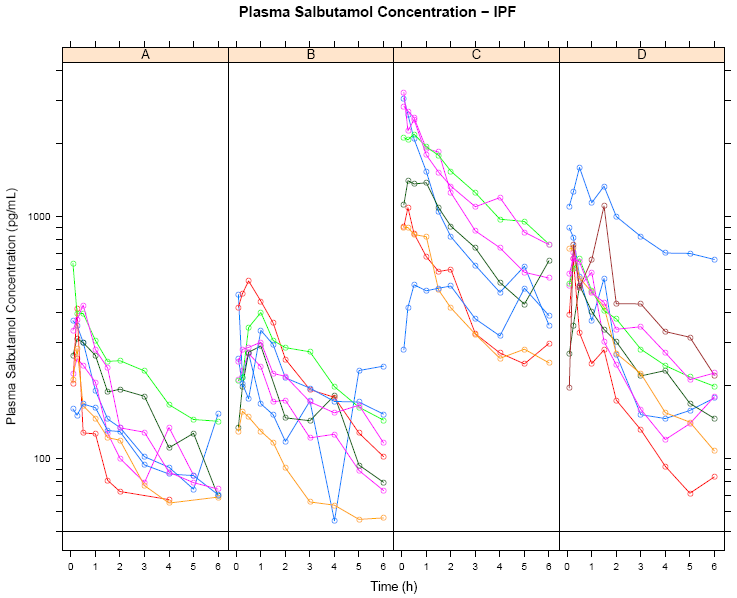


**Figure 12: Subject salbutamol plasma PK profiles by treatment group.** A: 50µg salbutamol was delivered at 1.5µm particle size via the STAG device; B: 50µg salbutamol was delivered at 6µm particle size via the STAG device; C: 2.5mg salbutamol was delivered via nebulisation; D: 400µg salbutamol was delivered via pMDI.

Figure 13: Amount of salbutamol in lungs vs the amount excreted in urine during the first 30min post dose

Figure 14 : Impulse Oscillometry parameters pre-dose, 60 minutes post-dose and 3 hours post-dose for each subject receiving: 50µg salbutamol delivered at 1.5µm particle size via the STAG; 50µg salbutamol delivered at 6µm particle size via the STAG; 2.5mg salbutamol delivered via nebulisation; 400µg salbutamol delivered via pMDI.

Figure 15: Pulmonary Function parameters pre-dose, 60 minutes post-dose and 3 hours post-dose for each subject receiving: 50µg salbutamol delivered at 1.5µm particle size via the STAG; 50µg salbutamol delivered at 6µm particle size via the STAG; 2.5mg salbutamol delivered via nebulisation; 400µg salbutamol delivered via pMDI.
